# *catena*-Poly[[[[2-(2-pyridyl-κ*N*)-1*H*-benzimidazole-κ*N* ^3^]copper(II)]-μ-l-methio­ninato-κ^3^ *N*,*O*:*O*′] perchlorate]

**DOI:** 10.1107/S1600536811014000

**Published:** 2011-04-29

**Authors:** Yan-Mei Lu, Xue-Yi Le

**Affiliations:** a Department of Applied Chemistry, South China Agricultural University, 510642 Guangzhou, Guangdong, People’s Republic of China; b Institue of Biomaterial, South China Agricultural University, 510642 Guangzhou, Guangdong, People’s Republic of China

## Abstract

The structure of the title compound, {[Cu(C_5_H_10_NO_2_S)(C_12_H_9_N_3_)]ClO_4_}_*n*_, has ortho­rhom­bic symmetry. The chain structure is constructed from square-pyramidally coordinated Cu^II^ atoms linked through l-methio­nate ligands. The chains propagate along the *a*-axis direction and are linked to perchlorate anions *via* N—H⋯O hydrogen bonds.

## Related literature

For the biological activity of benzimidazole derivatives and their metal complexes, see: Devereux *et al.* (2004 ▶, 2007 ▶); El-Sherif & Jeragh (2007 ▶). For metal complexes of l-α-amino acids, see: Lin *et al.* (2006 ▶), Yamauchi *et al.* (1992 ▶); Zhou *et al.* (2005 ▶).
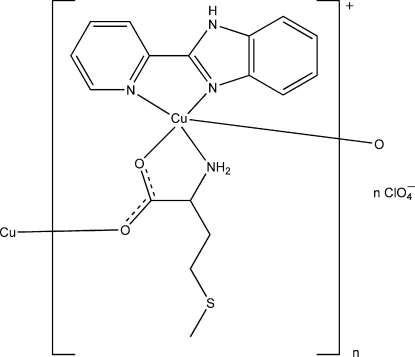


## Experimental

### 

#### Crystal data

[Cu(C_5_H_10_NO_2_S)(C_12_H_9_N_3_)]ClO_4_
*M* *_r_* = 506.41
Orthorhombic, 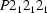

*a* = 6.9718 (4) Å
*b* = 11.8902 (6) Å
*c* = 24.7024 (13) Å
*V* = 2047.73 (19) Å^3^
*Z* = 4
Mo *K*α radiation
μ = 1.34 mm^−1^
*T* = 293 K
0.45 × 0.35 × 0.13 mm

#### Data collection

Bruker SMART 1000 CCD diffractometer
Absorption correction: multi-scan (*SADABS*; Sheldrick, 1996 ▶) *T*_min_ = 0.583, *T*_max_ = 0.845
12781 measured reflections
4464 independent reflections
3557 reflections with *I* > 2σ(*I*)
*R*_int_ = 0.035

#### Refinement

*R*[*F* ^2^ > 2σ(*F* ^2^)] = 0.042
*wR*(*F* ^2^) = 0.111
*S* = 1.05
4464 reflections
272 parameters
H-atom parameters constrained
Δρ_max_ = 0.45 e Å^−3^
Δρ_min_ = −0.28 e Å^−3^
Absolute structure: Flack (1983 ▶), 1874 Friedel pairs
Flack parameter: −0.001 (17)

### 

Data collection: *SMART* (Bruker, 2001 ▶); cell refinement: *SAINT-Plus* (Bruker, 2003 ▶); data reduction: *SAINT-Plus*; program(s) used to solve structure: *SHELXTL* (Sheldrick, 2008 ▶); program(s) used to refine structure: *SHELXTL*; molecular graphics: *ORTEP-3* (Farrugia, 1997 ▶); software used to prepare material for publication: *SHELXTL*.

## Supplementary Material

Crystal structure: contains datablocks I, global. DOI: 10.1107/S1600536811014000/ff2004sup1.cif

Structure factors: contains datablocks I. DOI: 10.1107/S1600536811014000/ff2004Isup2.hkl

Additional supplementary materials:  crystallographic information; 3D view; checkCIF report

## Figures and Tables

**Table 1:** Selected bond lengths (Å)

Cu1—O2^i^	2.272 (3)
Cu1—O1	1.929 (3)
Cu1—N1	1.996 (2)
Cu1—N3	2.023 (3)
Cu1—N4	1.985 (3)
O2—Cu1^ii^	2.272 (3)

Symmetry codes: (i) 
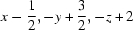
; (ii) 
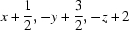
.

**Table 2:** Hydrogen-bond geometry (Å, °)

*D*—H⋯*A*	*D*—H	H⋯*A*	*D*⋯*A*	*D*—H⋯*A*
N2—H2a⋯O3^iii^	0.86	2.06	2.904 (6)	168
N4—H4a⋯O5^iv^	0.90	2.53	3.370 (7)	155
N4—H4b⋯O4	0.90	2.31	3.064 (7)	141

Symmetry codes: (iii) 
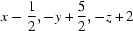
; (iv) 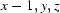.

**Fig. 1.**
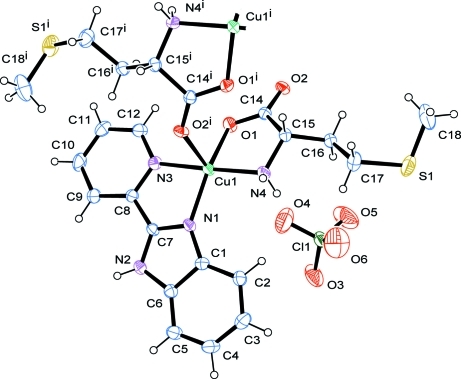
The molecular structure of the title compound, drawn with 30% probability displacement ellipsoids. Symmetry codes: (i) -0.5+x, 1.5-y, 2-z.

**Fig. 2.**
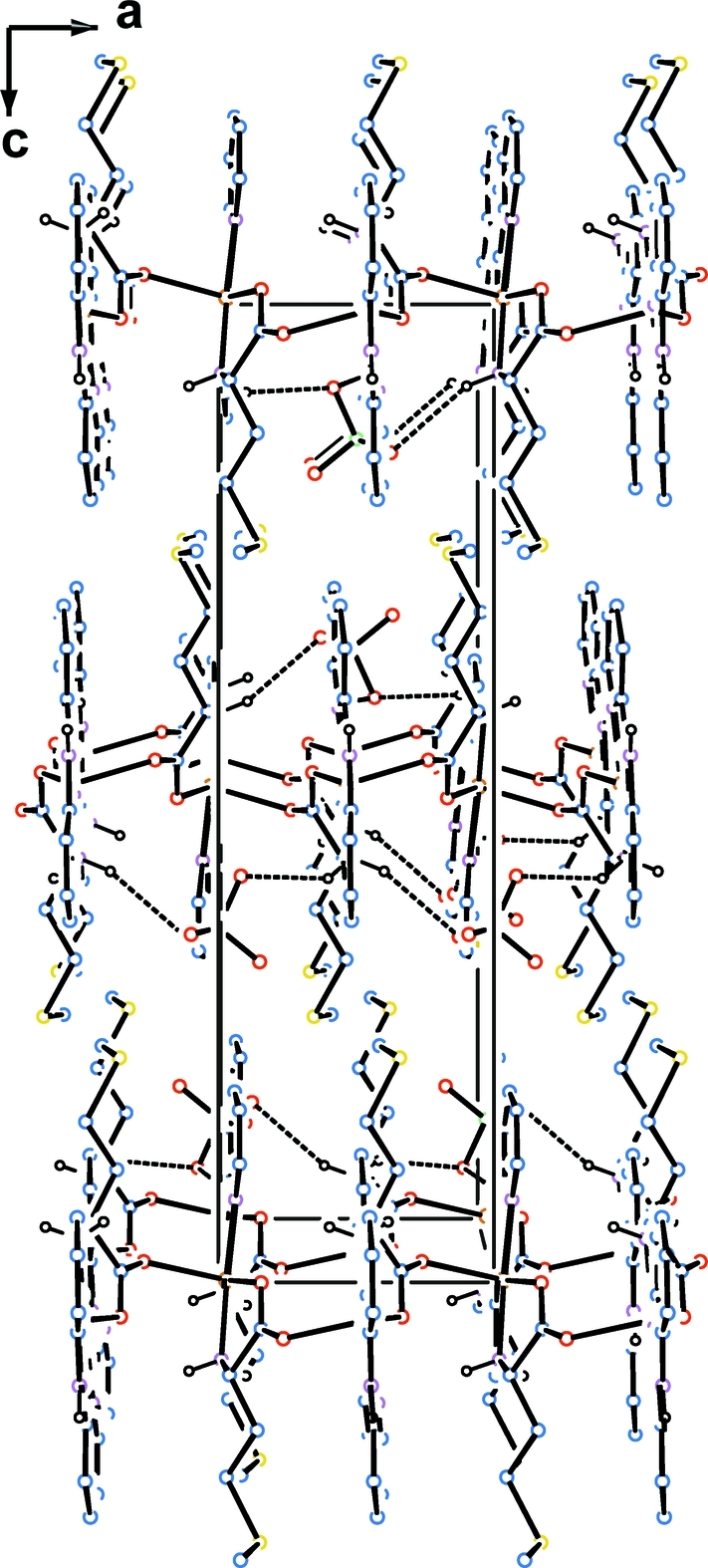
The crystal packing viewed along the b axis. Hydrogen bonds are drawn as dashed lines. Hydrogen atoms not involved in hydrogen bonding are omitted for clarity.

## supplementary crystallographic information

### Comment

In recent years, structure investigations of benzimidazole derivatives and their metal complexes have attracted an interest due to their antioxidant, antimycobacterium, antiparasitic activity and cytotoxicity (Devereux *et al.*, 2004, 2007; El-Sherif & Jeragh, 2007). Furthermore, L-α-amino acids are important biological ligands, taking flexible coordination modes with metal ions (Lin *et al.*., 2006, Yamauchi *et al.*, 1992, Zhou *et al.*, 2005). With L-α-amino acids being involved, the biological activities of complexes can be improved. We report herein the synthesis and crystal structure of the title complex.

The crystal structure of the title complex consists of [Cu(C_12_H_9_N_3_)(C_5_H_10_NO_2_S)]*_n_* polymeric chains (Fig. 2). The Cu(II) atom is in a slightly distorted square–pyramidal geometry (Fig. 1). The equatorial plane is occupied by two nitrogen atoms of 2-(2-pyridyl)benzimidazole ligand and one nitrogen atom and one oxygen atom of L-methionate ligand, while the apical position is occupied by another carboxylate oxygen atom from a symmetry-related neighboring L-methioninate ligand. The chains are connected by N—H···O hydrogen bonds to the perchlorate anions.

### Experimental

To a stirred ethanol solution (20 ml) containing 2-(2-pyridyl) benzimidazole (HPB) (0.098 g, 0.5 mmol) was added an aqueous solution(1 ml) of Cu(ClO_4_)_2_ 6H_2_O (0.188 g,0.5 mmol). An aqueous solution of L-Met(0.075 g, 0.5 mmol) and NaOH (0.020 g, 0.5 mmol) was then added to the mixture. After stirring continuously at 333 K for 1 h, the resulting green solution was filtered. The single crystals were obtained from the filtrate after two weeks (yield 67% based on Cu).

### Refinement

All hydrogen atoms were positioned geometrically and refined using a riding model, with C—H = 0.93–0.97 \%A, N—H = 0.86–0.9 \%A and with *U*_iso_(H) = 1.5 *U_eq_* (C) for methyl- H atoms, and 1.2 *U_eq_* (C,N) for the other hydrogen atoms.

### Crystal data

**Table:**

[Cu(C_5_H_10_NO_2_S)(C_12_H_9_N_3_)]ClO_4_	*F*(000) = 1036
*M**_r_* = 506.41	*D*_x_ = 1.643 Mg m^−^^3^
Orthorhombic, *P*2_1_2_1_2_1_	Mo *K*α radiation, λ = 0.71073 Å
Hall symbol: P 2ac 2ab	Cell parameters from 5099 reflections
*a* = 6.9718 (4) Å	θ = 2.4–26.5°
*b* = 11.8902 (6) Å	µ = 1.34 mm^−^^1^
*c* = 24.7024 (13) Å	*T* = 293 K
*V* = 2047.73 (19) Å^3^	Block, blue
*Z* = 4	0.45 × 0.35 × 0.13 mm

### Data collection

**Table:**

Bruker SMART 1000 CCD diffractometer	4464 independent reflections
Radiation source: fine-focus sealed tube	3557 reflections with *I* > 2σ(*I*)
graphite	*R*_int_ = 0.035
ω scans	θ_max_ = 27.1°, θ_min_ = 1.7°
Absorption correction: multi-scan (*SADABS*; Sheldrick, 1996)	*h* = −6→8
*T*_min_ = 0.583, *T*_max_ = 0.845	*k* = −15→14
12781 measured reflections	*l* = −30→31

### Refinement

**Table:**

Refinement on *F*^2^	Secondary atom site location: difference Fourier map
Least-squares matrix: full	Hydrogen site location: inferred from neighbouring sites
*R*[*F*^2^ > 2σ(*F*^2^)] = 0.042	H-atom parameters constrained
*wR*(*F*^2^) = 0.111	*w* = 1/[σ^2^(*F*_o_^2^) + (0.0626*P*)^2^] where *P* = (*F*_o_^2^ + 2*F*_c_^2^)/3
*S* = 1.05	(Δ/σ)_max_ = 0.001
4464 reflections	Δρ_max_ = 0.45 e Å^−^^3^
272 parameters	Δρ_min_ = −0.28 e Å^−^^3^
0 restraints	Absolute structure: Flack (1983), 1874 Friedel pairs
Primary atom site location: structure-invariant direct methods	Flack parameter: −0.001 (17)

### Special details

**Table:**

Geometry. All e.s.d.'s (except the e.s.d. in the dihedral angle between two l.s. planes) are estimated using the full covariance matrix. The cell e.s.d.'s are taken into account individually in the estimation of e.s.d.'s in distances, angles and torsion angles; correlations between e.s.d.'s in cell parameters are only used when they are defined by crystal symmetry. An approximate (isotropic) treatment of cell e.s.d.'s is used for estimating e.s.d.'s involving l.s. planes.
Refinement. Refinement of *F*^2^ against ALL reflections. The weighted *R*-factor *wR* and goodness of fit *S* are based on *F*^2^, conventional *R*-factors *R* are based on *F*, with *F* set to zero for negative *F*^2^. The threshold expression of *F*^2^ > σ(*F*^2^) is used only for calculating *R*-factors(gt) *etc*. and is not relevant to the choice of reflections for refinement. *R*-factors based on *F*^2^ are statistically about twice as large as those based on *F*, and *R*- factors based on ALL data will be even larger.

### Fractional atomic coordinates and isotropic or equivalent isotropic displacement parameters (Å^2^)

**Table:**

	*x*	*y*	*z*	*U*_iso_*/*U*_eq_	
Cu1	0.03097 (7)	0.92990 (4)	1.007152 (16)	0.03414 (14)	
S1	0.1487 (2)	0.72711 (14)	0.75829 (5)	0.0695 (4)	
C1	0.0206 (6)	1.1839 (3)	0.96479 (14)	0.0319 (8)	
C2	0.0198 (7)	1.1840 (3)	0.90774 (15)	0.0422 (10)	
H2	0.0161	1.1176	0.8879	0.051*	
C3	0.0246 (7)	1.2888 (4)	0.88271 (16)	0.0517 (11)	
H3	0.0244	1.2923	0.8451	0.062*	
C4	0.0297 (7)	1.3874 (4)	0.91190 (17)	0.0496 (11)	
H4	0.0311	1.4552	0.8932	0.060*	
C5	0.0329 (7)	1.3900 (3)	0.96696 (16)	0.0444 (10)	
H5	0.0390	1.4573	0.9861	0.053*	
C6	0.0265 (5)	1.2874 (3)	0.99268 (14)	0.0348 (8)	
C7	0.0303 (6)	1.1451 (3)	1.04985 (14)	0.0324 (8)	
C8	0.0452 (6)	1.0774 (3)	1.09845 (13)	0.0334 (8)	
C9	0.0441 (7)	1.1167 (4)	1.15117 (15)	0.0463 (10)	
H9	0.0334	1.1933	1.1584	0.056*	
C10	0.0590 (8)	1.0405 (4)	1.19261 (16)	0.0557 (13)	
H10	0.0574	1.0646	1.2284	0.067*	
C11	0.0767 (6)	0.9258 (4)	1.18046 (17)	0.0496 (11)	
H11	0.0878	0.8729	1.2080	0.059*	
C12	0.0774 (6)	0.8931 (4)	1.12754 (16)	0.0416 (10)	
H12	0.0885	0.8169	1.1196	0.050*	
C14	0.1548 (5)	0.7221 (3)	0.97297 (14)	0.0312 (8)	
C15	0.0420 (6)	0.7667 (3)	0.92357 (13)	0.0318 (8)	
H15	−0.0844	0.7307	0.9240	0.038*	
C16	0.1402 (6)	0.7342 (3)	0.87040 (15)	0.0374 (9)	
H16A	0.2677	0.7667	0.8697	0.045*	
H16B	0.1543	0.6530	0.8692	0.045*	
C17	0.0311 (7)	0.7726 (4)	0.81999 (15)	0.0542 (11)	
H17A	0.0217	0.8540	0.8201	0.065*	
H17B	−0.0980	0.7423	0.8210	0.065*	
C18	0.0818 (9)	0.5877 (5)	0.7533 (2)	0.088 (2)	
H18A	0.0984	0.5516	0.7878	0.132*	
H18B	0.1602	0.5508	0.7268	0.132*	
H18C	−0.0504	0.5831	0.7427	0.132*	
N1	0.0205 (4)	1.0973 (2)	1.00175 (11)	0.0331 (7)	
N2	0.0307 (5)	1.2578 (2)	1.04683 (12)	0.0381 (8)	
H2A	0.0330	1.3035	1.0738	0.046*	
N3	0.0626 (5)	0.9665 (3)	1.08662 (12)	0.0359 (8)	
N4	0.0118 (5)	0.8879 (2)	0.92955 (11)	0.0348 (7)	
H4A	−0.1048	0.9066	0.9167	0.042*	
H4B	0.1005	0.9257	0.9103	0.042*	
O1	0.1548 (4)	0.7856 (2)	1.01441 (10)	0.0413 (6)	
O2	0.2326 (4)	0.6305 (2)	0.96999 (10)	0.0381 (6)	
Cl1	0.50122 (15)	0.99416 (8)	0.86259 (4)	0.0468 (3)	
O3	0.5967 (8)	1.0975 (4)	0.8603 (2)	0.122 (2)	
O4	0.4179 (8)	0.9815 (5)	0.91477 (17)	0.1137 (18)	
O5	0.6217 (8)	0.9044 (4)	0.8494 (3)	0.129 (2)	
O6	0.3501 (8)	0.9903 (5)	0.8275 (2)	0.131 (2)	

### Atomic displacement parameters (Å^2^)

**Table:**

	*U*^11^	*U*^22^	*U*^33^	*U*^12^	*U*^13^	*U*^23^
Cu1	0.0428 (3)	0.0279 (2)	0.0317 (2)	0.0028 (2)	−0.00370 (19)	−0.00388 (17)
S1	0.0851 (10)	0.0913 (10)	0.0322 (6)	−0.0032 (8)	0.0089 (6)	0.0012 (6)
C1	0.027 (2)	0.0314 (18)	0.0379 (19)	−0.0007 (17)	−0.0005 (16)	0.0032 (14)
C2	0.054 (3)	0.039 (2)	0.0336 (18)	0.000 (2)	−0.003 (2)	−0.0036 (15)
C3	0.059 (3)	0.059 (3)	0.038 (2)	0.001 (3)	0.000 (2)	0.0104 (19)
C4	0.058 (3)	0.037 (2)	0.054 (2)	−0.005 (2)	−0.005 (2)	0.0116 (18)
C5	0.051 (3)	0.034 (2)	0.049 (2)	0.000 (2)	−0.003 (2)	0.0010 (17)
C6	0.0326 (19)	0.0346 (18)	0.0371 (18)	−0.0026 (16)	−0.0004 (18)	−0.0024 (15)
C7	0.036 (2)	0.0275 (18)	0.0336 (17)	−0.0022 (17)	−0.0004 (17)	−0.0055 (14)
C8	0.038 (2)	0.0324 (19)	0.0299 (17)	−0.0007 (19)	−0.0018 (15)	0.0003 (15)
C9	0.063 (3)	0.043 (2)	0.033 (2)	−0.006 (2)	−0.001 (2)	−0.0032 (16)
C10	0.070 (3)	0.071 (3)	0.0260 (19)	−0.005 (3)	−0.004 (2)	−0.0060 (19)
C11	0.052 (3)	0.061 (3)	0.036 (2)	−0.005 (2)	−0.0026 (18)	0.012 (2)
C12	0.046 (3)	0.037 (2)	0.042 (2)	−0.0045 (18)	−0.0055 (18)	0.0031 (17)
C14	0.029 (2)	0.033 (2)	0.0317 (18)	−0.0037 (17)	−0.0008 (15)	−0.0006 (16)
C15	0.030 (2)	0.0342 (19)	0.0311 (17)	0.0000 (17)	0.0007 (16)	−0.0035 (14)
C16	0.044 (2)	0.035 (2)	0.0331 (19)	0.0027 (18)	0.0022 (18)	−0.0028 (16)
C17	0.058 (3)	0.069 (3)	0.035 (2)	0.009 (3)	0.003 (2)	−0.003 (2)
C18	0.085 (4)	0.101 (5)	0.079 (4)	−0.011 (4)	0.012 (3)	−0.048 (4)
N1	0.0377 (17)	0.0278 (14)	0.0338 (15)	−0.0007 (12)	−0.0022 (15)	−0.0062 (11)
N2	0.047 (2)	0.0325 (17)	0.0349 (15)	−0.0035 (16)	0.0007 (16)	−0.0084 (12)
N3	0.0364 (19)	0.0374 (18)	0.0337 (16)	−0.0019 (14)	−0.0039 (14)	0.0003 (13)
N4	0.041 (2)	0.0294 (15)	0.0340 (15)	0.0083 (15)	−0.0022 (15)	−0.0002 (12)
O1	0.0508 (17)	0.0385 (15)	0.0347 (14)	0.0131 (13)	−0.0099 (13)	−0.0016 (12)
O2	0.0432 (16)	0.0257 (14)	0.0453 (16)	0.0071 (12)	−0.0008 (12)	−0.0022 (12)
Cl1	0.0479 (6)	0.0444 (5)	0.0481 (5)	−0.0055 (5)	0.0044 (5)	−0.0133 (4)
O3	0.154 (5)	0.079 (3)	0.132 (4)	−0.063 (3)	0.073 (3)	−0.055 (3)
O4	0.129 (4)	0.148 (4)	0.064 (3)	−0.044 (4)	0.026 (3)	−0.013 (3)
O5	0.120 (4)	0.074 (3)	0.192 (6)	0.029 (3)	0.052 (4)	−0.022 (3)
O6	0.104 (4)	0.176 (5)	0.114 (4)	0.002 (4)	−0.042 (3)	−0.016 (4)

### Geometric parameters (Å, °)

**Table:**

Cu1—O2^i^	2.272 (3)	C10—H10	0.9300
Cu1—O1	1.929 (3)	C11—C12	1.364 (6)
Cu1—N1	1.996 (2)	C11—H11	0.9300
Cu1—N3	2.023 (3)	C12—N3	1.339 (5)
Cu1—N4	1.985 (3)	C12—H12	0.9300
S1—C18	1.727 (6)	C14—O2	1.220 (4)
S1—C17	1.813 (4)	C14—O1	1.272 (4)
C1—N1	1.377 (4)	C14—C15	1.546 (5)
C1—C2	1.409 (5)	C15—N4	1.464 (4)
C1—C6	1.410 (5)	C15—C16	1.531 (5)
C2—C3	1.391 (6)	C15—H15	0.9800
C2—H2	0.9300	C16—C17	1.529 (6)
C3—C4	1.377 (6)	C16—H16A	0.9700
C3—H3	0.9300	C16—H16B	0.9700
C4—C5	1.360 (6)	C17—H17A	0.9700
C4—H4	0.9300	C17—H17B	0.9700
C5—C6	1.377 (5)	C18—H18A	0.9600
C5—H5	0.9300	C18—H18B	0.9600
C6—N2	1.383 (4)	C18—H18C	0.9600
C7—N1	1.319 (4)	N2—H2A	0.8600
C7—N2	1.341 (4)	N4—H4A	0.9000
C7—C8	1.449 (5)	N4—H4B	0.9000
C8—N3	1.356 (5)	O2—Cu1^ii^	2.272 (3)
C8—C9	1.384 (5)	Cl1—O6	1.365 (5)
C9—C10	1.372 (6)	Cl1—O5	1.397 (4)
C9—H9	0.9300	Cl1—O3	1.399 (4)
C10—C11	1.401 (6)	Cl1—O4	1.422 (4)
			
O1—Cu1—N4	84.05 (11)	O1—C14—C15	115.6 (3)
O1—Cu1—N1	155.45 (13)	N4—C15—C16	113.6 (3)
N4—Cu1—N1	100.58 (11)	N4—C15—C14	109.4 (3)
O1—Cu1—N3	92.99 (12)	C16—C15—C14	111.3 (3)
N4—Cu1—N3	176.82 (14)	N4—C15—H15	107.4
N1—Cu1—N3	81.63 (12)	C16—C15—H15	107.4
O1—Cu1—O2^i^	96.06 (11)	C14—C15—H15	107.4
N4—Cu1—O2^i^	95.67 (12)	C17—C16—C15	113.6 (4)
N1—Cu1—O2^i^	107.33 (11)	C17—C16—H16A	108.8
N3—Cu1—O2^i^	85.79 (11)	C15—C16—H16A	108.8
C18—S1—C17	102.9 (3)	C17—C16—H16B	108.8
N1—C1—C2	131.6 (3)	C15—C16—H16B	108.8
N1—C1—C6	109.2 (3)	H16A—C16—H16B	107.7
C2—C1—C6	119.2 (3)	C16—C17—S1	111.8 (3)
C3—C2—C1	116.4 (4)	C16—C17—H17A	109.3
C3—C2—H2	121.8	S1—C17—H17A	109.3
C1—C2—H2	121.8	C16—C17—H17B	109.3
C4—C3—C2	122.0 (4)	S1—C17—H17B	109.3
C4—C3—H3	119.0	H17A—C17—H17B	107.9
C2—C3—H3	119.0	S1—C18—H18A	109.5
C5—C4—C3	122.9 (4)	S1—C18—H18B	109.5
C5—C4—H4	118.5	H18A—C18—H18B	109.5
C3—C4—H4	118.5	S1—C18—H18C	109.5
C4—C5—C6	116.1 (4)	H18A—C18—H18C	109.5
C4—C5—H5	121.9	H18B—C18—H18C	109.5
C6—C5—H5	121.9	C7—N1—C1	105.9 (3)
C5—C6—N2	132.1 (3)	C7—N1—Cu1	111.6 (2)
C5—C6—C1	123.3 (3)	C1—N1—Cu1	142.2 (2)
N2—C6—C1	104.6 (3)	C7—N2—C6	107.9 (3)
N1—C7—N2	112.4 (3)	C7—N2—H2A	126.0
N1—C7—C8	120.7 (3)	C6—N2—H2A	126.0
N2—C7—C8	126.9 (3)	C12—N3—C8	118.5 (3)
N3—C8—C9	122.1 (3)	C12—N3—Cu1	126.9 (3)
N3—C8—C7	111.6 (3)	C8—N3—Cu1	114.1 (2)
C9—C8—C7	126.3 (3)	C15—N4—Cu1	109.6 (2)
C10—C9—C8	118.6 (4)	C15—N4—H4A	109.8
C10—C9—H9	120.7	Cu1—N4—H4A	109.8
C8—C9—H9	120.7	C15—N4—H4B	109.8
C9—C10—C11	119.3 (4)	Cu1—N4—H4B	109.8
C9—C10—H10	120.3	H4A—N4—H4B	108.2
C11—C10—H10	120.3	C14—O1—Cu1	116.9 (2)
C12—C11—C10	118.9 (4)	C14—O2—Cu1^ii^	132.4 (2)
C12—C11—H11	120.6	O6—Cl1—O5	106.9 (4)
C10—C11—H11	120.6	O6—Cl1—O3	111.8 (4)
N3—C12—C11	122.5 (4)	O5—Cl1—O3	112.1 (3)
N3—C12—H12	118.8	O6—Cl1—O4	104.9 (3)
C11—C12—H12	118.8	O5—Cl1—O4	112.1 (4)
O2—C14—O1	125.4 (3)	O3—Cl1—O4	108.9 (3)
O2—C14—C15	119.0 (3)		
			
N1—C1—C2—C3	−178.6 (4)	N4—Cu1—N1—C7	179.7 (3)
C6—C1—C2—C3	−0.1 (6)	N3—Cu1—N1—C7	−2.6 (3)
C1—C2—C3—C4	−0.1 (7)	O2^i^—Cu1—N1—C7	80.3 (3)
C2—C3—C4—C5	0.9 (8)	O1—Cu1—N1—C1	91.8 (5)
C3—C4—C5—C6	−1.4 (8)	N4—Cu1—N1—C1	−7.1 (5)
C4—C5—C6—N2	178.6 (5)	N3—Cu1—N1—C1	170.6 (4)
C4—C5—C6—C1	1.2 (6)	O2^i^—Cu1—N1—C1	−106.6 (4)
N1—C1—C6—C5	178.3 (4)	N1—C7—N2—C6	−1.9 (5)
C2—C1—C6—C5	−0.5 (6)	C8—C7—N2—C6	176.1 (4)
N1—C1—C6—N2	0.3 (4)	C5—C6—N2—C7	−176.9 (5)
C2—C1—C6—N2	−178.5 (4)	C1—C6—N2—C7	0.9 (5)
N1—C7—C8—N3	4.9 (6)	C11—C12—N3—C8	−0.4 (6)
N2—C7—C8—N3	−172.9 (4)	C11—C12—N3—Cu1	−172.4 (3)
N1—C7—C8—C9	−175.5 (4)	C9—C8—N3—C12	0.6 (6)
N2—C7—C8—C9	6.7 (7)	C7—C8—N3—C12	−179.8 (4)
N3—C8—C9—C10	−0.7 (7)	C9—C8—N3—Cu1	173.6 (3)
C7—C8—C9—C10	179.8 (4)	C7—C8—N3—Cu1	−6.8 (4)
C8—C9—C10—C11	0.6 (7)	O1—Cu1—N3—C12	−26.3 (4)
C9—C10—C11—C12	−0.4 (7)	N1—Cu1—N3—C12	177.7 (4)
C10—C11—C12—N3	0.3 (7)	O2^i^—Cu1—N3—C12	69.5 (3)
O2—C14—C15—N4	−163.0 (3)	O1—Cu1—N3—C8	161.3 (3)
O1—C14—C15—N4	18.2 (5)	N1—Cu1—N3—C8	5.4 (3)
O2—C14—C15—C16	−36.7 (5)	O2^i^—Cu1—N3—C8	−102.8 (3)
O1—C14—C15—C16	144.5 (3)	C16—C15—N4—Cu1	−147.4 (3)
N4—C15—C16—C17	−58.1 (5)	C14—C15—N4—Cu1	−22.4 (4)
C14—C15—C16—C17	178.0 (3)	O1—Cu1—N4—C15	17.0 (3)
C15—C16—C17—S1	−177.8 (3)	N1—Cu1—N4—C15	172.6 (3)
C18—S1—C17—C16	78.7 (4)	O2^i^—Cu1—N4—C15	−78.5 (3)
N2—C7—N1—C1	2.0 (5)	O2—C14—O1—Cu1	177.1 (3)
C8—C7—N1—C1	−176.1 (4)	C15—C14—O1—Cu1	−4.2 (4)
N2—C7—N1—Cu1	177.7 (3)	N4—Cu1—O1—C14	−7.3 (3)
C8—C7—N1—Cu1	−0.5 (5)	N1—Cu1—O1—C14	−109.8 (3)
C2—C1—N1—C7	177.2 (5)	N3—Cu1—O1—C14	173.8 (3)
C6—C1—N1—C7	−1.4 (4)	O2^i^—Cu1—O1—C14	87.8 (3)
C2—C1—N1—Cu1	3.9 (8)	O1—C14—O2—Cu1^ii^	−48.0 (5)
C6—C1—N1—Cu1	−174.8 (3)	C15—C14—O2—Cu1^ii^	133.4 (3)
O1—Cu1—N1—C7	−81.3 (4)		

Symmetry codes: (i) *x*−1/2, −*y*+3/2, −*z*+2; (ii) *x*+1/2, −*y*+3/2, −*z*+2.

### Hydrogen-bond geometry (Å, °)

**Table:**

*D*—H···*A*	*D*—H	H···*A*	*D*···*A*	*D*—H···*A*
N2—H2a···O3^iii^	0.86	2.06	2.904 (6)	168
N4—H4a···O5^iv^	0.90	2.53	3.370 (7)	155
N4—H4b···O4	0.90	2.31	3.064 (7)	141

Symmetry codes: (iii) *x*−1/2, −*y*+5/2, −*z*+2; (iv) *x*−1, *y*, *z*.
